# Household-level risk factors for water contamination and antimicrobial resistance in drinking water among households with children under 5 in rural San Marcos, Cajamarca, Peru

**DOI:** 10.1016/j.onehlt.2023.100482

**Published:** 2023-01-03

**Authors:** A.J. Larson, S. Haver, J. Hattendorf, G. Salmon-Mulanovich, M. Riveros, H. Verastegui, D. Mäusezahl, S.M. Hartinger

**Affiliations:** a Universidad Peruana Cayetano Heredia, Lima, Peru; b University of Washington, Seattle, United States; c Swiss Tropical and Public Health Institute, Allschwil, Switzerland; d University of Basel, Basel, Switzerland; e Institute for Nature, Earth and Energy at the Pontificia Universidad Católica del Perú, Lima, Peru

**Keywords:** Thermotolerant coliforms, Antimicrobial resistance, Domestic animals, Drinking water, One health

## Abstract

Household water contamination at point of use depends on human, animal and environmental factors embodying all aspects of a One Health approach. This study investigated the association between household factors, the presence of thermotolerant coliform, and the presence of antibiotic resistant bacteria in drinking water among 314 households with children under 5 in Cajamarca, Peru. This study analysed data from a baseline sampling of a randomized controlled trial, including household surveys covering household water management and factors such as household animals, as well as microbiological data from samples collected from drinking water. Data were analysed using generalized linear models. Drinking water samples collected from narrow-mouthed containers were less likely to be contaminated than samples collected from the faucet (OR = 0.55, *p* = 0.030) or wide mouthed containers. The presence of thermotolerant coliform was associated with owning farm birds, which increased the proportion of contamination from 42.2% to 59.1% (OR = 1.98, *p* = 0.017) and with animal waste observed in the kitchen area, which increased the prevalence of contamination from 51.4% to 65.6% (OR = 1.80, *p* = 0.024). Resistance to any antibiotic was higher among pig owners at 60%, relative to non-pig owners at 36.4% (OR = 1.97, *p* = 0.012) as well as households with free-roaming animals in the kitchen area at 59.6% compared to households without free-roaming animals at 39.7% (OR = 2.24, *p* = 0.035). Recent child antibiotic use increased the prevalence of trimethoprim-sulfamethoxazole resistance among *E. coli* isolates to 22.3% relative to 16.7% (OR = 3.00, *p* = 0.037). Overall, these findings suggest that water storage in a secure container to protect from in-home contamination is likely to be important in providing safe drinking water at point of use. In addition, transmission of thermotolerant coliform and AMR between domestic animals and human drinking water supplies is likely. Further research should explore transmission pathways and methods to support safe drinking water access in multi-species households.

## Highlights

• Household factors were associated with drinking water contamination and AMR.
• Narrow-mouthed or closed containers were less likely to be contaminated.
• Farm birds, and animal waste in the kitchen, were associated with contaminated water.
• Pigs, and free-roaming animals in the kitchen, were associated with AMR.
• Recent antibiotic use was associated with TMP-SMX resistance in water.

## 1 Introduction

Peru has undergone significant improvements in rural water access in the 21st century, but there are persistent disparities between urban and rural populations as well as documented vulnerabilities in the safety and proximity of rural water systems. WHO/UNICEF Joint Monitoring Programme has documented that the number of rural households which have access to a “basic service,” meaning that the household has an improved water source that can be collected within a 30 min round trip, has increased from 36.9% in 2000 to 58.7% in 2020 [1]. The Programa Nacional de Saneamiento Rural (National Program for Rural Sanitation) in Peru was created during this time [2] and there are ongoing programs to improve water infrastructure and treatment in rural areas, such as the Programa de Agua y Saneamiento Rural (Rural Sanitation and Water Program) [3] and the Agua Mas! program from the Ministerio de Desarrollo y Inclusión Social (Ministry of Development and Social Inclusion) [4]. Improvements in water supplies and sanitation have led to a steady decline in the national burdens of diarrheal disease in Peru in the 21st century [5]. Diarrheal diseases mortality in Peru fell from 8.9 deaths to 3.6 deaths per 100,000 people from 2000 to 2019, with a decline from 46.9 to 12.4 deaths per 100,000 children under 5 [6]. However, diarrheal disease morbidity and mortality remains a major preventable health problem countrywide with over 35 million cases and 1200 deaths from diarrheal illness documented in Peru in 2019 [6].

Despite these improvements, there are persistent discrepancies between urban and rural access to safe drinking water. In 2020, only 22.4% of rural households in Peru had access to a “safely managed service” for drinking water, meaning that the water is from an improved source, available on household premises and free of contamination, relative to 59.4% of urban households. These figures are similar to 2000 metrics (14.0% of rural households and 56.5% of urban households). In a study conducted by Rosa et al. (2014), they found that the majority of both urban and rural populations in Peru reported treating drinking water, but the proportion of households that had treated drinking water consistently during the sampling period was low in rural households (23.0%) relative to the urban population (67.1%) [7].

In addition, the importance of safe storage of water *within* the household to maintain the cleanliness of drinking water is well established in studies worldwide [[8], [9], [10]]. A combination of point-of-use treatment and safe storage, defined as a container with a narrow opening preventing contamination, achieve the greatest reduction in childhood diarrheal disease relative to other interventions, including treatment without safe storage [11]. However, other factors such as water storage, may obscure the effects of water treatment on contamination levels. Contamination of initially clean water through exposure to household storage was demonstrated in a study of households in peri-urban Lima, Peru, [12] and increased contamination in point-of-use water relative to source water has been demonstrated in studies globally [13]. Furthermore, contamination of treated drinking water can occur at the point-of-consumption from drinking vessels (Rufener, Mausezahl, Mosler, & Weingartner, 2010).

Gaps in clean, accessible water sources are relevant to discussions of antimicrobial resistance (AMR) and environmental contamination because inadequate drinking water systems can deliver antibiotic-resistant bacteria from local sources of contamination to humans [14]. Infections with antibiotic-resistant bacteria is associated with greater duration of illness and mortality compared to disease with antibiotic-susceptible bacteria, as well as rising health care costs [15]. AMR has been identified among clinical, community, animal and environmental domains in Peru [[16], [17], [18], [19], [20], [21], [22], [23], [24]]. Our previous work demonstrated that antibiotic resistant thermotolerant coliform contamination is present at high levels in drinking water in rural Cajamarca, but the pathways of contamination and factors which might promote AMR contamination of drinking water are not clear [21]. In general, AMR in the environment is promoted by release of AMR human and animal fecal waste into environmental reservoirs, as well as selective pressure from human and veterinary antimicrobial agents within clinical and community settings and in the environment [25]. The carriage and transmission of AMR in rural Latin American communities appears to be promoted by environmental transmission within the community as well as local antibiotic use [26]. Recent antibiotic use was associated with carriage of antibiotic-resistant *E. coli* among Peruvian children, while eating home-raised rather than “intensively antibiotic-raised market-purchased” chicken was found to have a protective effect [27].

The interaction of human and animal members in the household creates a quintessentially One Health network for water contamination and transfer of AMR. Household animals are associated with water contamination, including point of use water contamination, as well as diarrheal disease [[28], [29], [30], [31]]. Domestic animals in Peru are also valuable sources of economic security, enjoyment and emotional support [32,33]. Hence, this study explored whether specific animal species and environmental characteristics of the household are significantly associated with water contamination and AMR in the household water supply. We examined household risks and factors associated with drinking water contamination from a One Health lens incorporating human, animal and environmental factors contributing to drinking water contamination to further elucidate the pathways of environmental exposure to bacterial contamination and AMR.

## 2 Methods

### 2.1 Study site

The study was situated in rural San Marcos and Cajabamba in Cajamarca, Peru, part of the Andean region at approximately 2200–3900 m above sea level. Water is piped from central community reservoirs. These are typically large above ground tanks and water is delivered to individual households or courtyards with a standpipe where household members or neighbors can collect water in smaller buckets; inclusion criteria for this study required access to piped water in the home as described below.

### 2.2 Study sampling

This study was nested within a factor cluster-randomized controlled trial, which was conducted among households in 102 communities. Households with the following inclusion criteria were enrolled in the broader trial: at least one child < 1.5 years old in the household; solid fuels used as the primary fuel for cooking and/or heating; access to piped water within the household yard; no plans to move in the next two years; and not concurrently enrolled in another trial which may have affected outcomes [34]. A total of 320 households were selected for baseline and final measurements, including a socioeconomic survey detailing household characteristics, water testing for thermotolerant coliform counts and a survey of water use practices. Household sizes ranged from 3 to 10 people with a median of 5. In all cases, the member of the household completing the survey was the primary caretaker of the child. Biochemical and AMR testing for bacterial contamination was done with all water samples. Samples were collected and tested between November 2015 and April 2016, and socioeconomic surveys were performed between September 2015 and February 2016.

### 2.3 Sample collection

Surveys and water sample collection for 314 homes was performed by trained field staff during home visits. Surveys contained both closed and open-ended questions as well as direct observation to identify household factors such as animals in the kitchen environment [34]. Reported hand washing behaviors were collected from a subset of the households, but are not included here due to low sample sizes. Field staff collected 125 mL of water in sterile bottles from each household. The water samples were collected from the main water source from which the primary caregiver reported that the child in the household drank. Fieldworkers determined where the sample was collected from (including faucets and containers in the home) and classified the container from which the sample was taken; during the analysis, researchers categorized these containers as wide-mouthed (buckets, pots), and narrow-mouthed or containers that are typically poured from rather than dipped into (bottle, pitcher, tank, thermos, teapot, cup, plastic gallon jug). Samples were analysed for thermotolerant coliform counts in San Marcos within 8 h of collectione colonies per sample were sent to the Universidad Cayetano Peruana Heredia (UPCH) for further analysis. The field workers and the laboratory technician conducting the surveys, sampling and initial laboratory analysis were trained in survey administration and correct handling of samples.

### 2.4 Laboratory analysis

Household water samples were analysed within 8 h from collection for thermotolerant coliform count. Following incubation at 44 °C ± 0.5 °C from 14 to 16 h in a Lauryl Sulphate Tryptase broth, the membrane-filtration method of the Oxfam DelAgua water testing kit product code 14867 was used to isolate thermotolerant coliforms, a typical indicator of microbial water quality [35]. Five colonies per sample were transported to the UPCH for further analysis. All laboratory testing following field thermotolerant coliform counts was conducted in the Laboratorio de Enfermedades Entéricas y Nutrición from the Instituto de Medicina Tropical Alexander von Humboldt at UPCH.

Bacteria were identified using standard biochemical tests with conventional media [36]. Isolated bacteria were tested for resistance to a panel of antibiotics using a standard Kirby-Bauer disk diffusion procedure [37]. The antibiotics tested include nalidixic acid (30-μg disk), chloramphenicol (30-μg disk), nitrofurantoin (300-μg disk), ciprofloxacin (5-μg disk), gentamicin (10-μg disk), tetracycline (30-μg disk), trimethoprim-sulfamethoxazole (1.25/23.75-μg disk), amoxicillin-clavulanic acid (20/10-μg disk), ampicillin (10-μg disk), cefotaxime (30-μg disk), azithromycin (15-μg disk), and cefoxitin (30-μg disk; testing was performed for cefoxitin in *E. coli* isolates from only 31 households) according to Clinical and Laboratory Standards Institute guidelines [38]. Intermediate isolates were classified with susceptible isolates.

This study individually examines the antibiotics to which resistance was highest in a prior study: tetracycline, ampicillin, chloramphenicol, nalidixic acid, and trimethoprim-sulfamethoxazole [21]. Resistance to any antibiotic was also included, as well as multi-drug resistance. Resistance to “any antibiotic” included resistance to any individual antibiotic tested, while multi-drug resistance was defined as resistance to at least one antibiotic in three or more of the classes of antibiotics tested: quinolones (nalidixic acid or ciprofloxacin), phenicols (chloramphenicol), nitrofurans (nitrofurantoin), aminoglycosides (gentamicin), tetracyclines (tetracycline), folate pathway inhibitors (trimethoprim-sulfamethoxazole), penicillins (ampicillin or amoxicillin-clavulanic acid), macrolides (azithromycin), and cephalosporins (cephamycin or cefoxitin).

### 2.5 Statistical analysis

The analysis was performed using base R in R version 1.0.143. The associations between the presence or absence of thermotolerant coliforms and the likelihood of AMR within thermotolerant coliform samples by various metrics were modelled using generalized linear models and associations are presented as odds ratios. For the purpose of descriptive statistics, isolates labelled too numerous to count were represented as 385 colonies (1 above the documented maximum thermotolerant coliform count in this study). An appendix including a seasonal effects covariate was included. No adjustment was made for clustering at the community level as the intra-cluster correlation coefficients were very low (<0.1) for major outcome variables including presence of thermotolerant coliform and presence of AMR.

### 2.6 Ethics

This study was approved by the Cajamarca Regional Health Authority and the UPCH IRB (Ref 268–12-15), and was exempt from an ethical review by the Ethik Kommission Norwest- und Zentralschweiz. UPCH developed a collaborative agreement with community leaders and local authorities from the Cajamarca region prior to the implementation of the study. Written informed consent was obtained from each of the primary household respondents in the study.

## 3 Results

### 3.1 Respondents

In all cases, the primary respondent was the mother of the child under 5 years old. The age of respondents ranged from 15 to 46, with a median age of 26 years old.

### 3.2 Household factors

Water quality and water source container data were collected for 314 households, and surveys regarding household factors were conducted on a total of 306. Among all 314 households providing water samples, 114 (36.3%) provided the sample from a narrow-mouthed container, 54 (17.2%) from a wide-mouthed container and 87 (27.7%) provided the sample directly from the faucet (Table 1). Twenty-one (6.9%) of the 306 households that answered the socioeconomic survey data reported neither treating nor storing their drinking water; 105 (34.3%) reported storage but did not treat water, 175 (57.2%) stored and treated their water by boiling, and 5 (1.6%) households stored and treated water with chlorine or bleach (Table 1). Results from the socio-economic survey indicated 71 (23.2%) homes owned cows, 242 (79.1%) had farm birds such as chickens, 138 (45.1%) had pigs, 264 (86.3%) had guinea pigs (“cuy”) or rabbits, 171 (55.9%) had plow animals such as oxen, bulls, horses, donkeys and mules, and 99 (32.4%) had sheep, ram or goats (Table 1). Based on the observations of the fieldworker the kitchen environment contained open trash in 217 (70.9%) of the households, free-roaming animals in 124 (40.5%), animal fecal waste in 90 (29.4%), human waste (fecal waste, e.g. used diapers) in 43 (14.1%) of the households while flies or mosquitoes were present in 233 (76.1%) of households (Table 1). Application of antibiotics for child fevers, cough, or diarrhea in the previous 2 weeks was reported from 47 (15.4%) households (Table 1).

**Table 1:** Descriptive statistics of household factors among study sample households in rural Cajamarca, Peru.

Household factors	All households % [n/N]	All Households with *E. coli* % [n/N]
Variables observed at water sample collection (314 households):		
Water sample source:		
Faucet	27.7% [87/314]	36.8% [43/117]
Narrow containers	54.8% [172/314]	39.3% [46/117]
Wide containers	17.5% [55/314]	23.9% [28/117]
Variables reported in socioeconomic survey (306 households):^a^		
Reported treatment:		
No storage, No treatment	6.9% [21/306]	8.7% [10/115]
Storage, No treatment	34.3% [105/306]	34.8% [40/115]
Storage, Treatment by boiling	57.2% [175/306]	54.8% [63/115]
Storage, Treatment by chlorine/Bleach	1.6% [5/306]	1.7% [2/115]
Household animals		
Cows (Dairy/Beef)	23.2% [71/306]	26.1% [30/115]
Farm birds	79.1% [242/306]	87.8% [101/115]
Pigs	45.1% [138/306]	52.2% [60/115]
Cuy, Rabbits	86.3% [264/306]	87.8% [101/115]
Plow animals	55.9% [171/306]	66.1% [76/115]
Sheep, Ram, Goats	32.4% [99/306]	31.3% [36/115]
Kitchen area cleanliness (Presence of following, observed by researcher):		
Trash	70.9% [217/306]	68.7% [79/115]
Free-roaming animals	40.5% [124/306]	45.2% [52/115]
Animal waste^b^	29.4% [90/306]	36.5% [42/115]
Human waste^b^	14.1% [43/306]	15.7% [18/115]
Flies or mosquitoes	76.1% [233/306]	67.8% [78/115]
Child antibiotic use in 2 weeks prior to household survey		
Antibiotic use for fever, cough, or diarrhea	15.4% [47/306]	20.9% [24/115]

a Only households for which water samples were collected are included in this study. Of the 314 households for which water samples were collected and container observed at the point of collection, 306 also participated in the socioeconomic survey (SES) from which other variables were collected.

b Fecal waste, e.g. used diapers.

### 3.3 Household factors and bacterial contamination

#### 3.3.1 Water treatment, storage and contamination

Household water samples from narrow-mouthed containers were significantly less likely to be contaminated by thermotolerant coliforms (46.5%) compared to samples of from wide-mouthed containers (74.5%) and from a faucet (60.9%) (OR = 0.55, 95%CI [0.32, 0.94], *p* = 0.030) (Table 2). The finding that wide-mouthed containers were more likely to be contaminated (74.5%) (OR = 1.88, 95%CI [0.91, 4.04], *p* = 0.097) was marginally significant at *p* < 0.1. When this analysis was stratified by reported treatment type, narrow-mouthed containers from households reporting no treatment had significantly lower prevalence of thermotolerant coliform contamination (40.4%) relative to all other categories (OR = 0.21, 95%CI [0.21, 0.89], *p* = 0.024). (Table 2). Narrow-mouthed containers used in households with reported boiling were less likely to be contaminated than all other categories (48.7%) (OR = 0.61, 95%CI [0.34, 0.1.07], *p* = 0.085) and wide-mouthed containers used in houses with reported boiling were more likely to be contaminated than all other categories (80.8%) (OR = 2.70, 95%CI [0.99, 8.68], *p* = 0.068) with marginal significance at *p* < 0.1. Of note, while some of these containers (such as kettles) may have been used directly for boiling, other households may have boiled water and then transferred it into the recorded container. When a binary covariate representing season was introduced, narrow-mouthed containers were still associated with lower likelihood of contamination (OR = 0.63, 95%CI [0.36, 1.08], *p* = 0.094) while wide-mouthed containers were associated with a greater likelihood of contamination (OR = 2.09, 95%CI [0.99, 4.56], *p* = 0.057) but neither were significant at *p* < 0.05 (Appendix A, Table 3a). Self-reported household storage and water treatment (boiling or chlorine/bleach) were not significantly associated with the presence of any thermotolerant coliforms (Table 2).

**Table 2:** Associations between household factors and the presence of thermotolerant coliform in drinking water samples from households in rural Cajamarca, Peru.

Water storage and treatment		Coliform metrics			
		Households with thermotolerant coliform			
	Median [Mean]	% [n/N]	OR	95% CI	p
**All households**	2 [100.0]	55.4% [174/314]			
**Water sample source**					
Faucet	3 [81.1]	60.9% [53/87]			
Narrow containers	0 [93.4]	46.5% [80/172]	**0.55**	**[0.32,0.94]**	**0.030**
Wide containers	40 [150.3]	74.5% [41/55]	1.88	[0.91,4.04]	0.097
**Water sample source and treatment**^a^					
Faucet, All	3 [81.1]	60.9% [53/87]			
Narrow container, No treatment	0 [74.0]	40.4% [19/47]	**0.44**	**[0.21,0.89]**	**0.024**
Narrow container, Boiling	0 [107.1]	48.7% [57/117]	0.61	[0.34,1.07]	0.085
Narrow container, Chlorine/Bleach	5 [4.3]	66.7% [2/3]	1.28	[0.12,28.24]	0.84
Wide container, No treatment	40 [145.86]	68.9% [20/29]	1.43	[0.59,3.63]	0.44
Wide container, Boiling	51.5 [155.73]	80.8% [21/26]	2.70	[0.99,8.68]	0.068
**All Households participating in SES**^b^	2 [100.8]	55.6% [170/306]			
**Reported water treatment and self-reported storage**^c^					
No storage, No treatment	4 [45.6]	61.9% [13/21]			
Storage, No treatment	2 [93.5]	54.3 [57/105]	0.73	[0.27,1.88]	0.52
Storage, Boiling	2 [113.76]	54.9 [96/175]	0.75	[0.28,1.87]	0.54
Storage, Chlorine/Bleach	5 [32.0]	80.0 [4/5]	2.46	[0.29,52.8]	0.46
**Household animals, multivariate model of all animals**					
Cows (Dairy/Beef)	4 [114.8]	59.1% [42/71]	1.14	[0.65,2.01]	0.65
Farm birds	4 [110.8]	59.1% [143/242]	**1.97**	**[1.12,3.49]**	**0.020**
*No farm birds*	*0 [63.0]*	*42.2% [27/64]*			
Pigs	4 [106.4]	60.1% [83/138]	1.29	[0.80,2.10]	0.29
Cuy, Rabbits	2 [106.9]	56.4% [149/264]	1.14	[0.57,2.26]	0.71
Plow animals	3 [99.2]	57.9% [99/171]	1.18	[0.73,1.92]	0.50
Sheep, Ram, Goats	2 [87.0]	54.5% [54/99]	0.80	[0.73,1.92]	0.40
**Presence of the following in kitchen area, tested individually**					
Trash	2 [110.7]	55.3% [120/217]	0.96	[0.59,1.58]	0.89
Free-roaming animals	2.5 [123.1]	59.7% [74/124]	1.33	[0.84,2.11]	0.23
Animal waste	7.5 [140.4]	65.6% [59/90]	**1.80**	**[1.09,3.02]**	**0.024**
*No animal waste*	*1.0 [84.29]*	*51.4% [111/216]*			
Human waste	1 [72.6]	51.2% [22/43]	0.81	[0.43,1.56]	0.53
Flies or mosquitoes	2 [104.0]	52.8% [123/233]	0.62	[0.36,1.06]	0.083

a Responses for treatment were self-reported and did not correspond with observed behavior; for example, multiple households reported household treatment of water but were observed to provide a sample directly from the faucet. For this reason, faucet samples were not divided by reported treatment. No households providing water from a wide mouthed container reported treatment by chlorine/bleach.

b Only households for which water samples were collected are included in this study. Of the 314 households for which water samples were collected and container observed at the point of collection, 306 also participated in the socioeconomic survey (SES) from which other variables were collected.

c Storage was a binary,self-reported variable, while household water container was the container from which the fieldworker observed that the water was collected. Because no households reported treating water without also reporting storage of water, the variables for treatment and storage of water are presented as a combined analysis.

#### 3.3.2 Household environment and contamination

When household animals were modelled as a multivariate model, the presence of farm birds in the household significantly increased the prevalence of thermotolerant coliform contamination from 42.2% to 59.1% (OR = 1.92, 95%CI [1.09, 3.42], *p* = 0.025) (Table 2). No other subsistence animal was associated with the presence of thermotolerant coliforms, and the introduction of a binary covariate representing season did not significantly alter these results (Appendix A, Table 3a). Households with farm birds were more likely to be contaminated with *E. coli*: 21.9% with *E. coli* in households without farm birds in contrast to 41.7% (OR = 2.57, 95%CI [1.36, 5.13], *p* = 0.005) (Appendix, Table 1a). Households with pigs had greater prevalence of *Enterobacter* contamination, with *Enterobacter* identified in 4.9% of households without pigs and 8.0% in households with pigs (OR = 4.05, 95%CI [1.32, 14.50], *p* = 0.020) (Appendix, Table 1a). Households with plow animals had an increased likelihood of *E. coli* contamination from 28.9% to 44.4% (OR = 1.96, 95%CI [1.18, 3.29], *p* = 0.010) and decreased prevalence of both Enterobacter contamination (8.1% in homes without plow animals to 2.9% (OR = 0.31, 95%CI [0.09, 0.93], *p* = 0.045)), and Klebsiella contamination (11.9% in homes without plow animals to 5.3% (OR = 0.37, 95%CI [0.14, 0.89], p = 0.045)) (Appendix A, Table 1a).

The presence of animal waste significantly increased the prevalence of thermotolerant coliform contamination from 51.4% to 65.6% in the kitchen environment (OR = 1.80, 95%CI [1.09, 3.02], *p* = 0.024) (Table 2) as well as the prevalence of identifying *E. coli* from 33.8% to 46.7% (OR = 1.71, 95%CI [1.04, 2.83], *p* = 0.035) (Appendix A, Table 1a). The presence of flies or mosquitoes in the kitchen environment significantly lowered the prevalence of identifying *E. coli* from 50.7% to 33.5% (OR 0.49, 95%CI [0.29, 0.83], *p* = 0.0087) (Appendix A, Table 1a). When a binary seasonal covariate was included in this model, the presence of animal waste remained significant, but the presence of flies and mosquitoes was no longer significant at the *p* < 0.05 significance level (*p* = 0.059) (Appendix A, Table 3a).

### 3.4 Household factors and AMR

#### 3.4.1 Water treatment, storage and AMR

We assessed the presence of AMR in samples collected from households that reported storing their drinking water. Households reporting boiling and storage of water had significantly lower prevalence of identifying AMR to nalidixic acid at 4.8% relative to 30.0% in households reporting no water storage or treatment and 12.5% in households that reported storing but not treating water (OR: 0.12, 95% CI [0.018,0.73], *p* = 0.018) (Appendix A, Table 2a). Notably, households reporting boiling and storage had lower prevalence of AMR than households that reported storage but no treatment by all metrics included in this study, including ampicillin, trimethoprim-sulfamethoxazole, chloramphenicol, tetracycline and multi-drug resistance, although the odds ratio was only significant at p < 0.05 for nalidixic acid resistance (Table 3 and Appendix, Table 2a). Container type was not significantly associated with any metric of AMR.

**Table 3:** Associations between household factors and the presence of antibiotic resistance among *E. coli* isolates in drinking water samples from households in rural Cajamarca, Peru.

Water storage and treatment	Antibiotic resistance metrics							
	Resistance to any antibiotic				Resistance to 3 or more classes of antibiotics			
	% [n/N]	OR	95% CI	p	% [n/N]	OR	95% CI	p
**All households**	48.7% [57/117]				19.7% [23/117]			
Faucet^b^	53.5% [23/43]				18.6% [8/43]			
Narrow containers	52.2% [24/46]	0.94	[0.41,2.19]	0.90	23.9% [11/46]	1.38	[0.50,3.94]	0.54
Wide containers	35.7% [10/28]	0.48	[0.18,1.27]	0.15	14.2% [4/28]	0.73	[0.18,2.59]	0.64
**All households participating in SES**^a^	48.7% [57/117]				20.0% [23/115]			
**Reported treatment**								
No storage, No treatment	50.0% [5/10]				30.0% [3/10]			
Storage, No treatment	57.5% [23/40]	1.35	[0.33,5.60]	0.67	27.5% [11/40]	0.89	[0.20,4.67]	0.88
Storage, Treatment by boiling	42.9% [27/63]	0.75	[[0.19,2.95]	0.67	14.3% [9/63]	0.39	[0.088,2.05]	0.23
Storage, Treatment by chlorine/Bleach	50.0% [1/2]	1.0	[0.033,30.40]	1.0	0.0% [0/2]			
**Household animals, multivariate model of all animals**								
Cows (Dairy/Beef)	43.3% [13/30]	0.90	[0.46,1.74]	0.75	26.7% [8/30]	1.50	[0.69,3.16]	0.29
Farm birds	51.5% [52/101]	1.97	[0.83,5.28]	0.14	20.8% [21/101]	1.21	[0.43,4.78]	0.75
Pigs	60.0% [36/60]	**1.82**	**[1.04,3.20]**	**0.036**	26.7% [16/60]	1.86	[0.92,4.01]	0.094
*No pigs*	*36.4% [20/55]*							
Cuy, Rabbits	50.5% [51/101]	1.30	[0.54, 3.30]	0.56	20.8% [21/101]	1.15	[0.39,4.63]	0.82
Plow animals	46.1% [35/76]	0.84	[0.46, 1.52]	0.57	19.7% [15/76]	0.87	[0.42,1.83]	0.70
Sheep, Ram, Goats	41.7% [15/36]	0.74	[0.40, 1.35]	0.33	22.2% [8/36]	1.13	[0.53,2.32]	0.74
**Presence of the following in kitchen area, tested individually**								
Trash	53.2% [42/79]	1.78	[0.81, 4.05]	0.16	21.5% [17/79]	1.37	[0.51, 4.12]	0.55
Free-roaming animals	59.6% [31/52]	**2.24**	**[1.07, 4.80]**	**0.035**	25.0% [13/52]	1.77	[0.71, 4.54]	0.23
*No free-roaming animals*	*39.7% [25/63]*							
Animal waste	50.0% [21/42]	1.09	[0.51, 2.33]	0.83	21.4% [9/42]	1.15	[0.44, 2.91]	0.77
Human waste	44.4% [8/18]	0.82	[0.29, 2.24]	0.70	22.2% [4/18]	1.17	[0.31, 3.72]	0.80
Flies or mosquitoes	47.4% [37/78]	0.85	[0.39, 1.87]	0.70	17.9% [14/78]	0.68	[0.27, 1.80]	0.43
**Antibiotic use**								
Child antibiotic use in previous 2 weeks	51.6% [16/31]	0.86	[035, 2.13]	0.75	25.8% [8/31]	1.93	[0.66, 5.32]	0.21

a Only households for which water samples were collected are included in this study. Of the 314 households for which water samples were collected and container observed at the point of collection, 306 also participated in the socioeconomic survey (SES) from which other variables were collected.

#### 3.4.2 Household environment and AMR

In a multivariate model including all animals, the presence of pigs was associated with an increased prevalence of AMR to any antibiotic tested from 36.4% to 60% (OR = 1.82, 95% CI [1.04, 3.20], *p* = 0.036) (Table 3). Pigs were associated with an increased prevalence of resistance to ampicillin from 18.1% to 36.7% (OR = 1.91, 95% CI [1.02, 3.70], *p* = 0.047), trimethoprim-sulfamethoxazole from 9.1% to 26.7% (OR = 2.47, 95% CI [1.16, 5.91], *p* = 0.027), and tetracycline from 21.8% to 41.7% (OR = 1.83, 95% CI [1.01, 3.41], *p* = 0.049) (Appendix A, Table 2a).

Among the kitchen environment variable, the presence of free-roaming animals in the kitchen environment were associated with increased prevalence of AMR to any antibiotic from 39.7% to 59.6% (OR = 2.24, 95% CI [1.07, 4.80], *p* = 0.035) (Table 3), as well as resistance to tetracycline from 23.8% to 42.3% (OR = 2.35, 95% CI [1.06, 5.30], *p* = 0.037) (Appendix A, Table 2a). No other household animals or cleanliness metrics were significantly associated with AMR.

#### 3.4.3 Child antibiotic use and AMR

Child antibiotic use in the two weeks prior to the household survey was associated with increased prevalence of trimethoprim-sulfamethoxazole resistance among *E. coli* isolates in water samples from 16.7% to 22.3% (OR = 3.0, 95% CI [1.04, 8.39], p = 0.037) (Appendix A, Table 2a). Child antibiotic use was not significantly associated with any other metric of AMR.

## 4 Discussion

The findings of this study suggest that household factors including water containers, household animals, and child antibiotic use contribute to AMR contamination of drinking water among households in rural Peru. Households reporting water storage or treatment did not have significantly lower prevalence of thermotolerant coliform contamination. However, the type of container (wide or narrow mouthed) was found to be associated with contamination, where wide-mouthed containers were more likely to be contaminated. Free-roaming animals, animal waste in the kitchen, having chickens and pigs were also associated with increased contamination or AMR in the water supply, suggesting that household factors related to animals play a role in point of drinking water contamination. These findings suggest that in-home contamination and household animals contribute to drinking water contamination with AMR bacteria and can be used to guide further research to support safe treatment and storage of drinking water in rural Peru.

Consistent with other studies, our findings suggest that the type of container used for water storage may be a significant risk factor for drinking water contamination. It is interesting to note that while wide-mouthed containers did have elevated likelihood of contamination relative to samples from the faucet or other containers, this difference was only marginally significant. However, narrow-mouthed containers had a *lower* likelihood of contamination than either samples from the faucet or from wide-mouthed containers which was significant at *p* < 0.05. Controlling for seasonal confounding reduced the significance of the finding of lower contamination in narrow-mouthed containers from the p < 0.05 to *p* < 0.1 significance level but did not change the direction of these findings. This is broadly consistent with previous findings indicating that wide-mouthed containers are conducive to the practice of dipping hands or objects into water storage containers, which could re-contaminate water that has already been treated [39]. Kosek et al. (2008) found that poorly sealed or open containers were associated with a higher incidence of *Shigella* compared to properly sealed containers (Kosek et al., 2008). Multiple studies have shown decreased contamination with the introduction of safe storage (use of containers that reduce contamination by design, for example containers with covers and taps or spouts) compared to open containers [9,40]. The finding of a significant difference by container type is promising, particularly as this study did not provide any new containers or safe storage interventions. It may be possible to make recommendations at the household level for safe storage based on the existing resources of the household.

The finding that narrow-mouthed containers were significantly less likely to be contaminated than either wide-mouthed containers *or* samples from the faucet remains surprising for multiple reasons. First, while thermotolerant coliform levels might be expected to decrease over time in a correctly sealed container of water with no additional sources of contamination, previous literature generally suggests that water is likely to become more contaminated between source and point-of-use [9,13,41]. Point-of-use water treatment is one potential exception, and a previous study in another rural region of Peru found reduction in contamination with both safe storage and point-of-use treatment of water [39]. Effect modification by treatment was suspected, but our results further suggest that the finding of lower contamination in narrow-mouthed containers was *not* due to treatment between source and storage. The analysis of water source (wide container, narrow container or faucet) was stratified by reported treatment to identify possible effect modification between treatment and storage container. The findings of lower contamination in narrow-mouthed containers remained significant at *p* < 0.05 only for the subset of households that provided a sample from a narrow-mouthed container but reported no treatment. The overall direction of findings continued to indicate that narrow-mouthed containers had lower contamination than samples collected from the faucet or wide-mouthed containers, but households with treated water had *greater* odds of contamination within each container category overall. We suspect that the increase in contamination with treatment may be due to a combination of inadequate treatment or confounding by a hidden variable, in which households with greater overall contamination risk are also more likely to treat or to report treatment.

These findings also bring up an important limitation to the study. While fieldworkers described the container from which the water was provided, the treatment of water was a self-reported variable that may have been affected by reporter bias and was not necessarily consistent with the samples we received. For example, some houses reported household storage and/or treatment of drinking water, but fieldworkers noted that the sample was taken directly from the faucet. Since both storage and treatment were self-reported variables, the lack of significant findings related to self-reported storage and treatment may be due in part to survey error and discrepancy between the documented practices and those applied to collected samples. For example, some of the narrow containers were containers generally used to boil water, such as kettles. Participants may have been boiling water in those containers, thus reducing contamination levels, but not reporting that they were deliberately treating water by boiling. These findings highlight the need for detailed studies with active collaboration with research participants to gain an accurate understanding of how water is treated and stored, what sources of contamination exist within the household, and the real-world implications for drinking water.

AMR in drinking water contamination was significantly reduced among households that reported boiling water. Sub lethal heat stress has previously been demonstrated to lead to decreased levels of AMR in *E. coli* [42]*.* Boiling water lowered the odds of AMR to every antibiotic included in this study, including nalidixic acid (significant at *p* < 0.05), as well as tetracycline, ampicillin, chloramphenicol, and trimethoprim-sulfamethoxazole, as well as multi-drug resistance. These results suggest that water boiling may be a useful tool in reducing drinking water exposure to AMR elements, even if counts of thermotolerant coliform bacteria overall cannot be completely eliminated from the water supply by boiling. To the best of our knowledge this has not been described beyond the experimental study noted above [42]. The reduction in AMR could be alternatively explained by the idea that boiling eliminated an original population of bacteria present in source water that was then re-contaminated (e.g. from hand dipping) with another bacterial population with lower rates of AMR. To our knowledge there is no previous literature suggesting that in-home re-contamination of water is associated with lower AMR than contamination from external sources.

This study suggests a One Health connection between the microbiota of household animals and human drinking water exposures. We found that having farm birds, as well as the presence of animal waste in the kitchen environment, were both associated with the presence of thermotolerant coliforms in drinking water. We also found that having pigs, as well as presence of free-roaming animals in the kitchen environment, were associated with a greater likelihood of identifying AMR among isolated strains of *E. coli*. An important caveat to this finding is that the animal species in the kitchen environment was not recorded; the animals may have been dogs or cats rather than livestock, and could have been any kind of livestock. However, this is broadly consistent with a body of evidence supporting an association between household animals and microbial contamination of water [[28], [29], [30]] including contamination between source and point-of-use water [28]. Prior research has indicated that household birds, specifically, kept in close proximity to stored water increases the risk of contamination of point-of-use water [29]. Household animals may also be associated with greater risks of waterborne disease; a meta-analysis of domestic livestock and human diarrheal diseases indicated that in general, there is an association between domestic animal exposures and diarrheal disease in humans [31].

The path to reducing the risks of zoonotic disease in households raising animals must also consider the economic, social and emotional benefits of domestic and free-ranging animals and the demonstrated limitations of attempting to change livestock practices. The reasons for raising animals as free-ranging and allowing them into the home include the health, happiness and welfare of the animals and their growth; enjoyment of the animals' company; tradition; and the ability for children to grow up around animals and learn how to care for them [32]. Multiple studies evaluating poultry corralling to reduce exposure to domestic birds in Peru found that corralling livestock was not associated with less diarrheal disease [31,43]. Likewise, while pigs in rural Peru are frequently raised as free-roaming and may have exposure to human waste as a result, restraining them is not consistently associated with reduced rates of zoonotic disease and may limit the pigs' ability to forage and serve as “recyclers of waste” for the community [33]. As is typical of “One Health” questions, supporting human and animal health is not a straightforward endeavour and will require exploring what resources and support will allow human households to protect their water supply without compromising the care and use of their domestic animals.

Two inconsistent findings in this study warrant further exploration; we found ownership of plow animals associated with lower odds of identifying *Klebsiella* in drinking water (OR = 0.41, *p* = 0.042) while the presence of flies and/or mosquitoes in the kitchen environment was associated with lower odds of identifying *E. coli* in drinking water (OR = 0.49, *p* < 0.01). We believe that this finding is likely to be a spurious correlation, especially given the high prevalence of insects in the kitchen overall since most homes had open kitchens (including both outdoor kitchens and those with at least a window or door open to the outside); insects may have been very common and just not observed in a small sample of households. A key limitation of this study was the high number of tests of association performed on a relatively small sample of households, which is likely to generate occasional spurious findings. An alternative explanation is confounding factors; it is possible that households with insects in the kitchen were more proactive about protecting water supplies, and therefore had less contamination.

The use of antibiotics by children of the households in the study was only significantly associated with resistance to trimethoprim-sulfamethoxazole in drinking water. In our study, the type of antibiotic used recently by the children was not recorded. Nonetheless, trimethoprim-sulfamethoxazole was likely used in some cases. In a study of primary care physicians in peri-urban Lima, Peru, trimethoprim-sulfamethoxazole was found to be the most frequently preferred antibiotic to treat childhood dysentery [44]. Antibiotics are widely available in rural Peru and often given by parents to children without a prescription [45], which may be both a response to diarrheal disease and a contributing factor to AMR carriage. Kristiansson et al. (2009) found that child or family members' recent antibiotic use was associated with a greater odds of resistance among multiple antibiotics in a study of urban communities in Amazonian Peru (Kristiansson et al., 2009) [46]. Therefore, while the study design does not allow us to draw firm conclusions about transmission pathways, it is possible that trimethoprim-sulfamethoxazole resistance in this case is reflective of the use of antibiotics in the study population.

The limitations of this study include the possibility of recall bias for important measures, including antibiotic use by children. The water samples were collected over a time period of approximately 5 months and changing weather conditions or regional factors may have led to a bias in contamination depending on the time of surveying and/or sample collection. The time between the collection of the socioeconomic survey and the water samples (0–190 days, median 65 days) may have obscured the relationship between household factors and levels of water contamination, leading to type 2 error. In addition, households were considered individually, without divisions by water supply, neighbourhood, or other geographic factors that may have affected the results. We did not adjust for clustering at the community level due to low intra-cluster correlation coefficients, as described in the Methods section. Another limitation is that, as previously discussed, storage practice and treatment were assessed with self-reported measures while container type was observed. Self-reported metrics do not necessarily reflect the consistency or efficacy of a household's water storage and treatment practices, both paramount for disease prevention [7]. In this case, attempts to analyse data containing both self-reported and observed behaviors, which were sometimes contradictory (e.g. households reporting storage of water who then provided samples from the faucet), may have weakened the strength of our analyses. Water storage and treatment may have varied based on the availability and perceived cleanliness of piped water, in this particular cohort. For example, a household may consistently perform water treatment on surface water collected during a drought while drinking piped water, perceived as safe, straight from the faucet. Furthermore, water that is treated and then stored may also be perceived as safe, even though it may have been subjected to re-contamination through improper storage or the practice of dipping. Additionally, this study focused on the household environment and stated primary point of consumption within the home. Households may consume water from multiple sources [47] and it is possible that children may consume water from any number of sources outside the home, including schools, neighbors' homes or public spaces that may provide additional points of exposure to waterborne bacterial pathogens [48].

In the analysis of antibiotic resistance, a key limitation was that the analysis was limited to phenotypic data and did not incorporate a molecular genotyping approach. This was largely due to practical constraints of budget as well as sample storage and viability. Unfortunately, as a result we were unable to identify genetic sources of resistance as well as to use genetic markers to identify pathways of contamination. We hope that future research will be able to employ genetic markers to gain a fuller understanding of sources of contamination and antibiotic resistance.

Finally, one significant limitation is that only analyses using a binary outcome for the presence or absence of thermotolerant coliform are used due to the challenges of modelling thermotolerant coliform count with a large proportion of zero counts [49]. Factors affecting the level of thermotolerant coliform contamination are important, since higher thermotolerant coliform levels (specifically *E. coli*) in drinking water have previously been linked to higher rates of diarrheal disease [50]. However, neither presence nor level of thermotolerant coliform can be used as a direct indicator of diarrheal disease risk in a given household; rather, the presence or level of coliform is known to be generally associated with greater disease risk due to meta-analyses as well as the success of water quality interventions in reducing diarrheal disease [51]. We chose to focus on presence or absence of thermotolerant coliform both for clarity of analysis and because contamination is defined starting with a single thermotolerant coliform; WHO and Peruvian standards for coliform contamination in drinking water state that the acceptable level of detectable thermotolerant coliform is 0 CFU/mL [35,52].

## 5 Conclusions

Drinking water contamination with antibiotic-resistant bacteria in rural Cajamarca is associated with a variety of household factors including the kitchen environment and water handling, household animals, and child antibiotic use. Lessons for future safe drinking water initiatives include the importance of handwashing and supporting the use of clean, secure water containers to avoid in-home water contamination post water disinfection.

## Funding

This study received financial support from the 10.13039/501100008391UBS Optimus Foundation, the 10.13039/501100004828Grand Challenges Canada and Fogarty International Center of the National Institutes of Health under Award Number D43TW009375. The sponsors had no involvement in the study design, data collection and analysis, writing or the decision to submit the article for publication. The content is solely the responsibility of the authors and does not necessarily represent the official views of the National Institutes of Health.

## CRediT authorship contribution statement

**A.J. Larson:** Conceptualization, Methodology, Software, Formal analysis, Investigation, Writing - original draft, Writing - review & editing. **S. Haver:** Investigation, Writing - review & editing. **J. Hattendorf:** Methodology, Software, Formal analysis. **G. Salmon-Mulanovich:** Investigation, Writing - review & editing. **M. Riveros:** Methodology, Investigation, Resources, Writing – review & editing, Supervision. **H. Verastegui:** Project administration, Software, Data curation. **D. Mäusezahl:** Supervision, Project administration, Funding acquisition. **S.M. Hartinger:** Conceptualization, Methodology, Investigation, Writing – review & editing, Supervision, Funding acquisition.

## Declaration of Competing Interest

The authors declare that they have no known competing financial interests or personal relationships that could have appeared to influence the work reported in this paper.

## Figures and Tables

**Table 1a:** Associations between household factors and the presence of specific bacterial types isolated from drinking water samples from households in rural Cajamarca, Peru.

	Presence of specific bacterial types								
Household factors	*E. coli*			Enterobacter			Klebsiella		
	% [n/N]	OR	95% CI	% [n/N]	OR	95% CI	% [n/N]	OR	95% CI
**All households**	37.3% [117/314]			5.1% [16/314]			8.0% [25/314]		
**Water sample source:**									
Faucet	32.6% [14/43]			18.6% [8/43]			3.4% [3/87]		
**Narrow containers**	**26.7% [46/172]**	0.37**	**[0.21,0.64]**	7.0% [12/172]	6.45	[1.24,118.49]	7.0% [12/172]	2.10	[0.65,9.40]
**Wide containers**	50.9% [28/55]	1.06	[0.54,2.09]	5.5% [3/55]	4.96	[0.62,101.74]	**18.2% [10/55]**	6.22**	**[1.80,28.8]**
**All households participating in SES**^a^	37.6% [115/306]			5.2% [16/306]			8.2% [25/281]		
**Reported treatment:**									
No storage, No treatment	47.6% [10/21]			9.5% [2/21]			4.8% [1/21]		
Storage, No treatment	38.1% [40/105]	0.68	[0.26,1.76]	3.8% [4/105]	0.38	[0.068,2.85]	7.6% [8/105]	1.65	[0.28,31.47]
Storage, Boiling	36.0% [63/175]	0.62	[0.25,1.56]	5.1% [9/175]	0.52	[0.12,3.54]	8.6% [15/175]	1.88	[0.35,34.80]
Storage, Chlorine/Bleach	40.0% [2/5]	0.73	[0.083,5.33]	20.0% [1/5]	2.38	[0.097,31.76]	20.0% [1/5]	5.00	[0.17,146.37]
**Household animals**									
Cows (Dairy/Beef)	42.3% [30/71]	1.10	[0.62,1.95]	4.2% [3/71]	0.88	[0.19,3.03]	7.0% [5/71]	0.94	[0.29,2.55]
**Farm birds**	**41.7% [101/242]**	2.57**	**[1.36,5.13]**	5.8% [14/242]	1.97	[0.51,13.03]	7.9% [19/242]	0.81	[0.31,2.35]
***No farm birds***	***21.9% [14/64]***								
**Pigs**	43.5% [60/138]	1.41	[0.86,2.32]	**8.0% [11/138]**	4.05*	**[1.32,14.50]**	10.9% [15/138]	2.33	[0.97,5.89]
***No pigs***									
Cuy, Rabbits	38.3% [101/264]	1.02	[0.50,2.17]	4.9% [13/264]	0.44	[11,2.13]	8.3% [22/262]	0.94	[0.28,4.27]
**Plow animals**	**44.4% [76/171]**	1.96**	**[1.18,3.29]**	**2.9% [5/171]**	0.31*	**[0.09,0.93]**	**5.3% [9/171]**	0.37*	**[0.14,0.89]**
***No plow animals***	***28.9% [39/135]***			***8.1% [11/135]***			***11.9% [16/135]***		
Sheep, Ram, Goats	36.4% [36/129/]	0.69	[0.40,1.16]	3.0% [3/99]	0.53	[0.12,1.79]	7.1% [7/99]	0.99	[0.36,2.50]
**Cleanliness: Presence of the following in cooking or kitchen area**									
Trash	36.4% [79/217]	0.84	[0.51,1.40]	6.0% [13/217]	1.83	[0.57,8.11]	7.4% [16/217]	0.71	[0.31,1.73]
Free-roaming animals	41.9% [52/174]	1.36	[0.85,2.18]	4.8% [6/124]	0.87	[0.29,2.42]	7.3% [9/124]	0.81	[0.33,1.87]
**Animal waste**	**46.7% [42/90]**	1.71*	**[1.04,2.83]**	4.4% [4/90]	0.79	[0.22,2.34]	7.8% [7/90]	0.93	[0.35,2.22]
***No animal waste***	***33.8% [73/216]***								
Human Waste	41.9% [18/43]	1.23	[0.63,2.36]	2.3% [1/43]	0.39	[0.02,2.02]	4.7% [2/43]	0.51	[0.080,1.81]
**Flies or mosquitoes**	**33.5% [78/233]**	0.49**	**[0.29,0.83]**	5.6% [13/233]	1.38	[0.43,6.14]	7.7% [18/233]	0.79	[0.33,2.1]
***No flies or mosquitoes***	***50.7% [37/73]***								

^**⁎**^**denotes p** **<** 0.05, **denotes *p* **<** **0.01.**

a Only households for which water samples were collected are included in this study. Of the 314 households for which water samples were collected and container observed at the point of collection, 306 also participated in the socioeconomic survey (SES) from which other variables were collected.

**Table 2a:** Associations between household factors and the presence of antibiotic resistance against individual antibiotics among *E. coli* isolated from drinking water samples from households in rural Cajamarca, Peru.

	Antibiotic resistance														
	Ampicillin			Trimethoprim-sulfamethoxazole			Chloramphenicol			Nalidixic acid			Tetracycline		
Household factors	% [n/N]	OR	95% CI	% [n/N]	OR	95% CI	% [n/N]	OR	95% CI	% [n/N]	OR	95% CI	% [n/N]	OR	95% CI
**All households**	28.2% [33/117]			17.9% [21/117]			11.1% [13/117]			9.4% [11/117]			32.5% [38/117]		
**Water sample source:**															
Faucet	32.6% [14/43]			18.6% [8/43]			16.3% [7/43]			7.0% [3/43]			27.9% [12/43]		
Narrow containers	28.3% [13/46]	0.82	[0.33,2.02]	19.6% [9/46]	1.06	[0.37,3.13]	6.5% [3/46]	0.36	[0.07,1.39]	8.7% [4/46]	1.27	[0.26,6.78]	41.3% [19/47]	1.82	[0.75,4.50]
Wide containers	21.4% [6/28]	0.56	[0.18,1.65]	14.3% [4/28]	0.73	[0.18,2.59]	10.7% [3/28]	0.62	[0.12,2,46]	14.3% [4/28]	2.22	[0.45,12.10]	25.0% [7/28]	0.86	[0.28,2.51]
**All SES households**^a^	27.8% [32/115]			18.3% [21/115]			11.3% [13/115]			9.6% [11/115]			32.2% [37/115]		
**Reported treatment:**^a^															
No storage, No treatment	40.0% [4/10]			30.0% [3/10]			20.0% [2/10]			30.0% [3/10]			40.0% [4/10]		
Storage, No treatment	37.5% [15/40]	0.90	[0.22,4.00]	25.0% [10/40]	0.77	[0.18,4.13]	20.0% [8/40]	1.0	[0.20,7.48]	12.5% [5/40]	0.33	[0.065,1.92]	42.5% [17/40]	1.11	[0.27,4.91]
Storage, Boiling	19.0% [12/63]	0.35	[0.086,1.56]	12.7% [8/63]	0.34	[0.075,1.81]	4.8% [3/63]	0.20	[0.029,1.69]	**4.8% [3/63]**	0.12*	**[0.018,0.73]**	25.4% [16/63]	0.51	[0.13,2.21]
Storage, Chlorine/Bleach	50.0% [1/2]			0.0% [0/2]			0.0% [0/2]			0.0% [0.2]			0.0% [0/2]		
**Household animals**															
Cows	26.7% [8/30]	0.94	[0.45,1.89]	20.0% [6/30]	1.23	[0.53,2.73]	13.3% [4/30]	1.18	[0.43,2.96]	13.3% [7/85]	1.29	[0.46,3.38]	36.7% [11/30]	1.26	[0.64, 2.47]
Farm birds	28.7% [29/101]	1.25	[0.49,3.81]	18.8% [19/101]	1.09	[0.37, 4.38]	11.9% [12/101]	1.43	[0.40,11.64]	9.9% [10/101]	1.32	[0.55,3.91]	33.7% [34/101]	1.40	[0.56,4.24]
**Pigs**	**36.7% [22/60]**	1.91*	**[1.02,3.70]**	**26.7% [16/60]**	2.47*	**[1.16, 5.91]**	11.7% [7/60]	0.99	[0.42,2.38]	13.3% [8/60]	2.44	[0.89,8.41]	**41.7% [25/60]**	1.83*	**[1.01, 3.41]**
***No pigs***	**18.1% [10/55]**			***9.1% [5/55]***									**21.8% [12/55]**		
Cuy. Rabbits	28.7% [29/101]	1.07	[0.41,3.30]	18.8% [19/101]	0.97	[0.31, 3.98]	11.9% [12/101]	1.48	[0.40,12.15]	8.9% [9/101]	0.47	[0.13,2.05]	33.7% [34/101]	1.29	[0.50, 3.97]
Plow animals	28.9% [22/76]	1.21	[0.63,2.37]	15.8% [12/76]	0.69	[0.33,1.46]	11.8% [9/76]	1.04	[0.42,2.81]	10.5% [8/76]	1.25	[0.46,3.95]	31.6% [24/76]	0.89	[0.48,1.68]
Sheep, Ram, Goats	22.2% [8/36]	0.74	[0.36,1.44]	16.7% [6/36]	0.97	[0.42,2.09]	13.9% [5/36]	1.19	[0.47,2.83]	11.% [4/36]	1.26	[0.44,3.36]	33.3% [12/36]	1.04	[0.54, 1.95]
**Cleanliness: Presence of the following in cooking or kitchen area**															
Trash	30.4% [24/79]	1.53	[0.63,4.02]	20.3% [16/79]	1.57	[0.56, 5.17]	12.7% [10/79]	1.59	[0.45, 7.46]	10.1% [8/79]	1.24	[0.33, 5.93]	32.9% [26/79]	1.11	[0.48, 2.68]
**Free-roaming animals**	30.8% [16/52]	1.31	[0.57,2.97]	17.3% [9/52]	0.89	[0.33, 2.30]	13.5% [7/52]	1.48	[0.46, 4.89]	9.6% [5/52]	1.01	[0.28, 3.56]	**42.3% [22/52]**	2.35*	**[1.06, 5.30]**
***Free-roaming animals***													***23.8% [15/63]***		
Animal waste	21.4% [9/42]	0.59	[0.23,1.41]	16.7% [7/42]	0.84	[0.29, 2.23]	11.9% [5/42]	1.10	[0.31, 3.54]	11.9% [5/42]	1.51	[0.41, 5.34]	38.1% [16/42]	1.52	[0.68, 3.40]
Human waste	33.3% [6/18]	1.37	[0.44, 3.91]	11.1% [2/18]	0.51	[0.077, 2.02]	5.6% [1/18]	0.42	[0.02, 2.34]	0% [0/18]	Not performed.		27.8% [5/18]	0.78	[0.23, 2.27]
Flies or mosquitoes	26.9% [21/78]	0.87	[0.37,2.11]	19.2% [15/78]	1.23	[0.45, 3.73]	10.3% [8/78]	0.73	[0.23, 2.58]	10.3% [8/78]	1.30	[0.35, 6.19]	30.8% [24/78]	0.82	[0.36, 1.91]
**Child antibiotic use in 2 weeks prior to SES**															
**Antibiotic use**	29.0% [9/31]	1.09	[0.38, 2.85]	**22.3% [7/31]**	3.00*	**[1.04, 8.39]**	9.7% [3/31]	0.29	[0.015, 1.57]	16.1% [5/31]	1.48	[0.31, 5.64]	32.3% [10/31]	1.07	[0.39, 2.73]
***No antibiotic use***				***16.7% [14/84]***											

*denotes p **<** 0.05, **denotes p **<** **0.01.**

a Only households for which water samples were collected are included in this study. Of the 314 households for which water samples were collected and container observed at the point of collection, 306 also participated in the socioeconomic survey (SES) from which other variables were collected.

**Table 3a:** Associations between household factors and the presence of thermotolerant coliform in drinking water samples from households in rural Cajamarca, Peru with seasonal covariate.

Water storage and treatment		Coliform metrics			
		Households with thermotolerant coliform			
	**Median [Mean]**	**% [n/N]**	**OR**	**95% CI**	**p**
**All households**	2 [100.0]	55.4% [174/314]			
**Water sample source**					
Faucet	3 [81.1]	60.9% [53/87]			
Narrow containers	0 [93.4]	46.5% [80/172]	0.63	[0.36,1.08]	0.094
Wide containers	40 [150.3]	74.5% [41/55]	2.09	[0.99,4.56]	0.057
**All Households participating in SES**^a^	2 [100.8]	55.6% [170/306]			
**Reported water treatment with binary seasonal covariate**					
No storage, No treatment	4 [45.6]	61.9% [13/21]			
Storage, No treatment	2 [93.5]	54.3 [57/105]	0.73	[0.27,1.91]	0.53
Storage, treatment by boiling	2 [113.76]	54.9 [96/175]	0.73	[0.27,1.84]	0.51
Storage, Treatment by chlorine/Bleach	5 [32.0]	80.0 [4/5]	2.86	[0.34,61.9]	0.34
**Household animals**					
Cows (Dairy/Beef)	4 [114.8]	59.1% [42/71]	1.13	[0.64, 2.00]	0.67
Farm birds	4 [110.8]	59.1% [143/242]	**1.92**	**[1.09,3.42]**	**0.025**
*No farm birds*	*0 [63.0]*	*42.2% [27/64]*			
Pigs	4 [106.4]	60.1% [83/138]	1.33	[0.82,2.17]	0.25
Cuy, Rabbits	2 [106.9]	56.4% [149/264]	1.07	[0.53,2.13]	0.85
Plow animals	3 [99.2]	57.9% [99/171]	1.20	[0.74,1.95]	0.47
Sheep, Ram, Goats	2 [87.0]	54.5% [54/99]	0.80	[0.48,1.34]	0.40
**Kitchen area cleanliness (Presence of the following)**					
Trash	2 [110.7]	55.3% [120/217]	0.94	[0.57,1.55]	0.81
Free-roaming animals	2.5 [123.1]	59.7% [74/124]	1.30	[0.82,2.08]	0.26
Animal waste	7.5 [140.4]	65.6% [59/90]	**1.80**	**[1.08,3.03]**	**0.025**
*No animal waste*	*1.0 [84.29]*	*51.4% [111/216]*			
Human waste	1 [72.6]	51.2% [22/43]	0.78	[0.41,1.50]	0.46
Flies or mosquitoes	2 [104.0]	52.8% [123/233]	0.59	[0.34,1.01]	0.059

a Only households for which water samples were collected are included in this study. Of the 314 households for which water samples were collected and container observed at the point of collection, 306 also participated in the socioeconomic survey (SES) from which other variables were collected.

## Data Availability

Data will be made available on request.
